# Injection of immunoglobulin in the treatment process of children with severe pneumonia

**DOI:** 10.12669/pjms.35.4.83

**Published:** 2019

**Authors:** Liping Chen, Xuehai Qi, Ruiying Li, Xudong Wang, Baohai Shi, Qingmei Meng

**Affiliations:** 1 Liping Chen, Department of Pediatrics, Tai’an City Central Hospital, Tai’an, 271000, China; 2 Xuehai Qi, Department of Pediatrics, Tai’an Maternal and Child Health Hospital, Tai’an, 271000, China; 3 Ruiying Li, Department of Gynecology, Tai’an City Central Hospital, Tai’an, 271000, China; 4 Xudong Wang, Department of Pediatrics, Tai’an City Central Hospital, Tai’an, 271000, China; 5 Baohai Shi, Department of Pediatrics, Tai’an City Central Hospital, Tai’an, 271000, China; 6 Qingmei Meng, Department of Pediatrics, Tai’an City Central Hospital, Tai’an, 271000, China

**Keywords:** Immune globulin, Children, Treatment, Severe pneumonia

## Abstract

**Objective:**

To observe the effect of intravenous infusion of immune globulin in the treatment of children with severe pneumonia.

**Methods:**

Ninety-eight children with severe pneumonia who received treatment in our hospital between April 2015 and December 2016 were selected, and they were grouped into a control group and an treatment group, 49 each group. The control group received conventional treatment. The treatment group was additionally treated with immune globulin on the basis of conventional treatment. The humoral immune indicators were detected using immunoturbidimetric assay, and the inflammatory reaction indicators were detected using enzyme-linked immunoabsorbent kit. The overall efficacy, clinical symptoms, humoral immunity and inflammatory response were compared between the two groups.

**Results:**

The total effective rate of the treatment group was significantly higher than that of the control group (P<0.05). The cough, rale and fever of the treatment group disappeared faster than those of the control group, the relief of cardiac failure was also faster in the treatment group (P<0.05). The immunoglobulin G (Ig G), Ig A and Ig M of patients in the two groups compared before treatment, and no significant difference was found (P>0.05). The Ig G level of the treatment group was higher than that of the control group after treatment (P<0.05). The comparison of Ig A and Ig M between the two groups indicated no significant difference (P>0.05). The two groups had no significant differences in the content of tumor necrosis factor (TNF)-α, C-reactive protein (CRP), soluble intercellular adhesion molecule (SICAM)-1 and interferon (IFN)-γ (P>0.05) before treatment, but the content of the treatment group was significantly higher than that of the control group (P<0.01).

**Conclusion:**

Immunoglobulin was significantly effective in the adjuvant treatment of children with severe pneumonia, and it can rapidly improve the improvement of symptoms, enhance immune function and inhibit inflammatory reaction; therefore it is worth promotion.

## INTRODUCTION

Severe pneumonia is a common emergency and severe respiratory disease in the department of pediatric internal medicine and a main cause for death of hospitalized infants in China.^1^,^2^ Severe pneumonia in children which is usually caused by respiratory syncytial virus infection can weaken the immune function of children; featured by acute onset and severe disease condition, it can lead to death if treatment is not performed timely.^3^ Severe pneumonia in children is characterized by systemic poisoning, which can not only severely damage the respiratory system, but also cause different degrees of damages to other systems and a series of symptoms such as water and electrolyte balance disorders and myocarditis.^4^ It develops quickly and usually results in death of children due to the delayed or ineffective treatment.^5^,^6^ For the treatment of severe pneumonia in children, it is necessary to correct hypoxia, carry out active treatment and prevent complications.

In recent years, some studies have suggested that severe pneumonia in children is related to the disorder of immune function, and inhibition of humoral immune response and insufficient secretion of immunoglobulin (Ig) are important reasons for the development and poor prognosis of severe pneumonia.^7^-^9^ Ig for intravenous injection is a blood product purified from a large number of healthy human plasma, and its main ingredient is protein. Ig has functions of immunoregulation and immune substitution; therefore it has good treatment efficacy in diseases related to the immune system of children.^10^ Moreover it has been reported that children with severe pneumonia are prone to have systemic transitional inflammatory response which can stimulate the body to produce a large amount of pro-inflammatory cytokines, mediate local inflammatory reaction, cause severe inflammatory damages to tissues and organs, induce cytokine cascade reaction.^11^ This study emphatically analyzed the efficacy of intravenous injection of Ig in the adjuvant treatment of children with severe pneumonia and its influence on humoral immunity and inflammatory state.

## METHODS

This study enrolled 98 children with severe pneumonia who were treated in our hospital in the period from April 2015 to December 2016. The children developed different levels of dyspnea, cyanosis and cough. X-ray films showed flaky shadows in the lung of children, and moreover the lung texture increased and dry and wet rale appeared. The 98 children were randomly divided into treatment group and a control group according to the random table method, with 49 cases in each group. There were 26 males and 23 females in the treatment group; their age ranged from 1 to 10 years (mean (5.6±2.7) years); 14 children had central failure, 9 children had acute cerebral edema, 11 children had microcirculation, 8 children had acidosis, and 7 children had gastrointestinal dysfunction. There were 27 males and 22 females in the control group; their age aged from one to 10 years (mean (5.3±2.9) years); 13 children had central dysfunction, 8 had acute cerebral edema, 12 had microcirculation dysfunction, 9 had acidosis, and 7 had gastrointestinal dysfunction. The results were comparable as there were no statistical differences in gender, age, disease severity and physical condition between the two groups (P>0.05).

### Inclusive and exclusive criteria

Children who satisfied the diagnostic criteria of severe pneumonia mentioned in Guidelines for Management of Community Acquired Pneumonia in Children (revised),^12^ aged one month ~ 14 years, hospitalized in intensive care unit for more than 24 h, had complete clinical data, and were confirmed having bacterial infection by sputum or throat swab culture rather than other pathogen infection such as virus, fungi, mycoplasma and chlamydia were included. But children who have received anticoagulant therapy before entering intensive care unit, had blood system disease or congenital coagulation system abnormality, or had malignant tumor, hypertension, diabetes or severe liver and renal diseases were excluded from this study. This study has been reviewed and approved by the ethics committee of our hospitals, and the family members of the children signed informed consent.

### Treatment method

The control group received the traditional treatment, i.e. intravenous injection of azithromycin injection at a dose of 1 mg/kg/d, for 7 days. Moreover treatment which could relieve cough and asthma and aerosol inhalation were used.

Patients in the treatment group was given intravenous injection of Ig at a dose of 0.4 g•kg^-1^•d^-1^ for three days.

### (1) Evaluation of overall treatment effect:^13^

Treatment was evaluated as significantly effective if the symptoms and vital signs completely disappeared, urine volume significantly increased, and X-ray film suggested absorption of pulmonary lesions and strong heart sound one week after treatment. Treatment was evaluated as effective if the symptoms and vital signs improved, urine volume increased, and X-ray film suggested partial absorption of pulmonary lesions one week after treatment. Treatment was evaluated as ineffective if the symptoms and vital signs had no improvement or even aggravated, urine volume had no obvious increase, and X-ray film suggested no absorption of pulmonary lesions. The overall effective rate could be calculated using the formula: overall effective rate=(number of significantly effective cases+number of effective cases)/total number of cases*100%.

### (2) Improvement of clinical symptoms

The improvement of clinical symptoms including cough, lung rale, fever and cardiac failure was compared.

### (3) Detection of serum humoral immunity and inflammatory reaction indexes

Firstly 3~5 mL of peripheral venous blood was collected from each patient before treatment and in the 1^st^ week after treatment. After 15~30 min, the blood samples were centrifuged at 3000 r/min for 10 min. Then the upper layer of serum was carefully collected. The content of IgG, IgA and IgM was detected using immunoturbidimetry. The content of tumor necrosis factor (TNF)-α, C-reactive protein (CRP), soluble intercellular adhesion molecule (SICAM)-1 and interferon (IFN)-γ were detected using enzyme-linked immunoabsorbent kit.

### Statistical analysis

Statistical processing was made using SPSS ver. 20.0. Categorical data were expressed by cases and processed by Chi-square test. Measurement data were expressed by Mean±SD and processed by two-independent sample t test. Difference was thought statistically significant if P<0.05.

## RESULTS

The overall effective rate of the treatment group was 95.92%, higher than 83.67% in the control group, and the difference had statistical significance (P<0.05, Table-I). The cough, rale and fever of the treatment group disappeared faster than those of the control group, and the relief of cardiac failure was also faster in the treatment group (P<0.05); and the differences were statistically significant (P<0.05, Table-II).

**Table I:** Comparison of clinical efficacy between the two groups [n(%)].

Group	Significantly effective	Effective	Ineffective	Total effective rate
Treatment group	32(65.31)	15(30.61)	2(4.08)	47(95.92)
Control group	25(59.02)	16(32.65)	8(16.33)	41(83.67)
X^2^				9.337
P				<0.05

**Table II:** Comparison of disappearance time of symptoms between the two groups.

Group	Disappearance time of cough	Disappearance time of rale	Disappearance time of fever	Improvement time of cardiac failure
Treatment group	5.81±0.22	5.24±0.29	1.51±0.60	1.10±0.65
Control group	8.61±0.72	7.50±0.83	3.96±0.02	2.36±0.93
t	15.662	13.254	7.568	5.506
P	<0.05	<0.05	<0.05	<0.05

There was no significant difference in the levels of Ig G, Ig A and Ig M between the two groups before treatment (P>0.05). The levels of Ig G, Ig A and Ig M in the two groups after treatment were higher than those before treatment, and the level of Ig G in the treatment group was higher than that in the control group (P<0.05). The differences of the levels of IgA and IgM between the two groups had no statistical significant (P>0.05, Table-III).

**Table III:** Changes of Ig of the two groups before and after treatment.

Group		Ig G	Ig A	Ig M
Treatment group	Before	7.20±0.52	0.46±0.13	0.83±0.26
	After	12.21±0.82*^#^	0.56±0.14*	0.87±0.31*
Control group	Before	7.19±0.51	0.47±0.12	0.82±0.27
	After	11.20±0.79*	0.55±0.13*	0.86±0.30*

Note:

* means P<0.05 compared to before treatment in the same group;

# indicated P<0.05 comparing to the control group.

### Changes of content of inflammatory factors

Before treatment, no differences were found in the content of TNF-α, CRP, sICAM-1 and IFN-γ between the two groups (P<0.05). After treatment, the content of TNF-α, CRP, sICAM-1 and IFN-γ of the two groups significantly decreased (P<0.05), and moreover the content of the treatment group was significantly lower than that of the control group (P<0.05, Table-IV).

**Table IV:** Changes of content of inflammatory factors in serum before and after treatment.

Group		TNF-α (g/L)	CRP (mg/L)	sICAM-1 (ng/L)	IFN-γ (ng/L)
Treatment group	Before	4.58±0.61	59.43±7.75	9.28±1.46	12.54±1.86
	After	1.77±0.26*^#^	15.67±1.93*^#^	3.43±0.51*^#^	4.65±0.83*^#^
Control group	Before	4.65±0.72	60.15±7.86	9.34±1.17	12.91±2.22
	After	2.56±0.37*	25.51±4.57*	4.96±0.78*	7.75±0.94*

Note:

* indicated P<0.05 comparing to before treatment in the same group;

# indicated P<0.05 comparing to the control group.

## DISCUSSION

Pneumonia is a common disease in children in China, with the highest incidence in winter and spring. Lung inflammation of children is caused by inhalation of amniotic fluid, pathogen infection and allergic reaction. The symptoms of pneumonia in children are cough, fever, dyspnea and moist rale. A study shows that the progression of infantile pneumonia to severe pneumonia is related to the low specific and non-specific immune function, and the incidence of complications is high, which poses a great threat to children’s life and health.^14^ At present, symptomatic support, antimicrobial and antiviral therapy have been widely used in clinic, but the effect is not satisfactory.

The immunoglobulin used in this study was prepared from healthy plasma. Ig G is the main component of immunoglobulin, accounting for about 90%, which can prevent infection and enhance the resistance of human body. Immunoglobulin also contains a large number of specific antibodies which can resist viruses, bacteria and various pathogenic microorganisms.^15^,^16^ A study has suggested that the differentiation and maturity of B lymphocytes is inhibited and the synthesis and secretion of corresponding Ig significantly reduces in the development of severe pneumonia, which can affect the killing effect of humoral immune response on pathogens.^17^ In children with pneumonia, the secretion of Ig G is the most obvious, the secretion of IgA is slightly reduced, but the secretion of Ig M has no significant changes. Ig injection is mainly used to supplement Ig G in clinical treatment, which is consistent with the humoral immune characteristic of children with severe pneumonia that Ig G content is relatively low. It can effectively correct the pathological state of children with severe pneumonia whose humoral immune response is inhibited and Ig G synthesis and secretion is insufficient.^18^ The results of this study showed that the level of Ig G in the treatment group was significantly higher than that in the control group, and the improvement of Ig A and Ig M levels was also greater than that in the control group, which indicated that intravenous injection of Ig could rapidly increase the level of Ig in children with severe pneumonia, enhance immune function and accelerate the clearance of pathogenic microorganisms in the respiratory tract, which was consistent with the results of Zhou et al.^19^ The results of the present study showed that the total effective rate of the treatment group was significantly higher than that of the control group, and the disappearance time of cough, rales and fever and improvement time of cardiac failure of the treatment group were shorter than those of the control group. It indicated that Ig adjuvant therapy could improve the treatment effect and prognosis of severe pneumonia and was conducive to the relief of clinical symptoms and recovery of signs, which was consistent with the research results of previous studies.^20^,^21^

Activation of systemic inflammatory response is an important pathological feature in children with severe pneumonia. A large number of inflammatory factors such as TNF-α, CRP, sICAM-1 and IFN-γ are secreted into the blood circulation. TNF-α can mediate the cascade reaction of inflammation and kill pathogens by increasing the release of proteolytic enzymes and neutrophil degranulation.^22^ CRP is an acute phase reactant which is synthesized by the liver; pathogen infection of human body can significantly promote the synthesis and secretion of CRP. sICAM-1 is a kind of adhesion molecule, which can mediate the aggregation and metastasis of inflammatory cells, promote the recruitment of inflammatory cells and the activation of inflammatory reaction in local lesions.^23^ IFN-γ is secreted by NK cells and T cells, which can activate monocyte macrophages and significantly enhance the phagocytosis function of pathogen.^24^ The results of this study showed that the serum levels of TNF-α, CRP, sICAM-1 and IFN-γ in the treatment group were significantly lower than those in the control group, suggesting that Ig adjuvant treatment could significantly reduce the synthesis and secretion of multiple inflammatory factors to inhibit inflammatory reaction and improve the effect of clinical treatment.

## CONCLUSION

In the treatment of children with severe pneumonia, intravenous injection of Ig on the basis of conventional treatment has a sound effect. The therapy can effectively improve the treatment effect, accelerate the improvement of symptoms, enhance the immune functions of body and inhibit inflammatory reaction. But the size of samples was small and the observation time was short; hence the safety of medication has not been considered, which needs to be further studied in larger sample size in the future.
